# The roles of vocal and visual interactions in social learning zebra finches: A video playback experiment

**DOI:** 10.1016/j.beproc.2016.12.009

**Published:** 2017-06

**Authors:** Lauren M. Guillette, Susan D. Healy

**Affiliations:** School of Biology, University of St Andrews, UK

**Keywords:** Copying, Foraging, Social interaction, Social learning, Video playback, Zebra finch

## Abstract

The transmission of information from an experienced demonstrator to a naïve observer often depends on characteristics of the demonstrator, such as familiarity, success or dominance status. Whether or not the demonstrator pays attention to and/or interacts with the observer may also affect social information acquisition or use by the observer. Here we used a video-demonstrator paradigm first to test whether video demonstrators have the same effect as using live demonstrators in zebra finches, and second, to test the importance of visual and vocal interactions between the demonstrator and observer on social information use by the observer. We found that female zebra finches copied novel food choices of male demonstrators they saw via live-streaming video while they did not consistently copy from the demonstrators when they were seen in playbacks of the same videos. Although naive observers copied in the absence of vocalizations by the demonstrator, as they copied from playback of videos with the sound off, females did not copy where there was a mis-match between the visual information provided by the video and vocal information from a live male that was out of sight. Taken together these results suggest that video demonstration is a useful methodology for testing social information transfer, at least in a foraging context, but more importantly, that social information use varies according to the vocal interactions, or lack thereof, between the observer and the demonstrator.

## Highlights

• Zebra finches learn social information about foraging from video demonstrators.
• Live streaming has the same effect on observers’ behaviour as live demonstration.
• Silent video has the same effect on observers’ behaviour as live demonstration.
• Observers did not copy the video with sound.
• Observers did not copy when there was a decoy male present.

## 1 Introduction

Learning through interactions with, or observations of, other individuals occurs across a large range of animals and behaviours, including foraging, mate-choice, tool manufacture and use, nest-site location, and nest-material selection (i.e. social learning, e.g., Heyes, 1994, Heyes, 2012; Auersperg et al., 2014, Guillette et al., 2016, Kendal et al., 2015, Loukola et al., 2012, Trompf and Brown, 2014, Vakirtzis, 2011). Many of these studies are conducted in the laboratory using a live observer–demonstrator paradigm, which allows control of *who* provides information to the observer. The trade-off to using this paradigm is that even after extensive training the performance of the demonstrator and thus the social information provided to the observer can vary substantially from trial-to-trial. The housing and training of the demonstrator individuals can also be time consuming. Video playback may provide a way to standardize the demonstration and to reduce the total number of animals needed, as well as providing repeatability and stimulus control ([Bibr bib0030][Bibr bib0135]).

Video stimuli has been show to elicit natural behaviour in some animals. Chimpanzees (*Pan troglodytes*) and budgerigars (*Melopsittacus undulates*) yawn more in response to videos of conspecifics yawning than to control videos in which conspecifics were engaged in other behaviours (Campbell and de Waal, 2011, Gallup et al., 2015). Male zebra (*Taeniopygia guttata*) and Bengalese finches (*Lonchura striata*) sing directed song to video presentations of female conspecifics (Ikebuchi and Okanoya, 1999) and female zebra finches will perform courtship display to videos of male conspecifics (Swaddle et al., 2006). Gloomy octopus (*Octopus tetricus*) readily approached and touched the video screen when presented with a crab video, and reduced their activity, a natural response for this solitary species, in response to a video of a conspecific (Pronk et al., 2010). Videos of ‘audience’ hens (*Gallus domesticus*) potentiate alarm calls produced in the presence of a predator model (Evans and Marler, 1991) and male Jacky dragons (*Amphibolurus muricatus*) produce aggressive displays in response to videos of conspecific males (Ord et al., 2002). In some of these cases there is no qualitative difference in the response to live versus video stimuli (e.g. Evans and Marler, 1991, McQuoid and Galef, 1993, Ord et al., 2002, Rieucau and Giraldeau, 2009), however, in other cases an attenuated (e.g. Ikebuchi and Okanoya, 1999) or enhanced response to video stimuli was reported (Swaddle et al., 2006). In the latter, an enhanced response of female courtship to videos compared to live males may be the due to the non-interactive nature of video demonstration. The females who saw the non-interactive videos may have increased their courtship displays to elicit a response from the video male.

In addition to soliciting natural behaviour videos playback have also been used, albeit to a lesser extent, to examine social learning. In a sub-set of these studies the degree to which animals learn from live demonstrators relative to what they learn from demonstrators on video was compared: budgerigars copy the actions of a live or video demonstrators in a two-action test (Heyes and Saggerson, 2002, Mottley and Heyes, 2003) and Burmese red jungle fowl (*Gallus gallus spadecius*) copy foraging choices of live and video demonstrators (McQuoid and Galef, 1993, McQuoid and Galef, 1992). In the former test, the audio that accompanied the video demonstration was always played, while in the latter study one condition included both video and audio and a second condition included video only. The observers used social information only when both video and audio were available. In other tests of social learning of mate-choice in Japanese quail (*Coturnix japonica*) and tool manufacture and use in chimpanzees sound was not necessary for social learning to occur (Ophir and Galef, 2003, Price et al., 2009). Taken together these results suggests that social learning across species and/or context may require different types/quality of social information/interaction between the observer and the demonstrator.

The zebra finch is a gregarious species that takes well to laboratory conditions. Perhaps this is why the zebra finch has become a popular model species for studying song learning, neurobiology, mate choice, animal personality and cognition (Healy et al., 2010, Schuett and Dall, 2009, Zann, 1996). It is also a useful species to test hypotheses regarding the conditions under which animals should use social information. For example, developmentally stressed zebra finch chicks have weaker social associations with their parents compared to control chicks that were not stressed during development (Boogert et al., 2014) and early-life stress results in juveniles learning from unrelated adults (Farine et al., 2015). Female zebra finches, when faced with making a novel foraging decision, will copy the choice of live male demonstrators, whereas male zebra finches observing male demonstrators do not (Guillette and Healy, 2014). Males do, however, use social information in other situations: first-time nest-builders will copy the nest material choice of familiar, but not unfamiliar males (Guillette et al., 2016). Furthermore, zebra finches also pay attention to vocal information: not only are they vocal learners (Zann, 1996) with both males and females preferring their fathers songs over unfamiliar songs (Riebel et al., 2002), female zebra finches’ preference for their pair-bonded mate declines when auditory cues from their mate are masked by white noise (Swaddle et al., 2006) and zebra finch pairs will vocal duet at the nest (Elie et al., 2010).

In the current set of experiments we had two objectives. The first was to determine whether video demonstrators have the same effect on the behaviour of observers as do live demonstrators. To do this we followed the methodology of a previous study in which we found that zebra finch females, when faced with making a novel foraging decision, copied the choice of live male demonstrators while males did not copy anyone (Guillette and Healy, 2014), but in which we replaced live demonstrators with demonstrators presented on video. We presented a live video of male demonstrators feeding from one, but not a second novel feeder, to females or males on a thin-film-transistor (TFT) screen. TFT screens have constant illumination and can accommodate the higher flicker-fusion frequency of birds (Galoch and Bischof, 2007, Ikebuchi and Okanoya, 1999). As the demonstrator was in the same room but was visually occluded from the observer, the observer and the demonstrator could vocally interact, but the only visual information available to the observer was provided by the TFT screen. If video presentations are to be useful as a methodology for investigating social learning the live streaming video demonstration should produce the outcome described in Guillette and Healy (2014) in which female observers copied the foraging decisions of male demonstrators while male observers did not copy the foraging decisions of male demonstrators.

Second, we wanted to test the importance of social interactions in the transfer of information from a knowledgeable demonstrate to a naïve observer. To do this we ran three more manipulations in which we varied the type of social interaction that was available. Different observers were used in each experiment. In Experiment 2 the videos, with audio, of the male demonstrators recorded in Experiment 1 were played back to female observers. Thus, the observers could see and hear the demonstrators, but they lacked the vocal interaction in Experiment 1. In Experiment 3 we presented the demonstrator videos to female observers without the audio. In Experiment 4, we presented the same videos to female observers coupled with placing a decoy male in the room with which the females could vocally interact but could not see. This decoy male was not the male on the video. Therefore, in Experiment 4, the female observers could interact vocally with a male but he was not the demonstrator. In comparing the results of these four experiments we can determine both whether video playback is a valid experimental tool for assessing social learning about foraging and what social interactions are important for this learning.

## 2 Methods

### 2.1 Subjects

The subjects were either bread at the University of Andrews or purchased from a local breeder. All birds were housed in cages of same-sex individuals (8–10 individuals per cage, 100 × 50 × 50 cm or 15–30 individual per cage, 140 × 71 × 122 cm) and kept on a 14:10 light:dark cycle with temperature at ∼20 °C and humidity at ∼50%. Birds were given free access to mixed seeds, water (vitamin-supplemented 3 days per week), cuttle bone, oystershell, and vitamin block and fresh spinach (3 × per week). Each cage had several perch sizes and types and floors were covered with pressed wood pellets. At the end of the experiment birds were returned to the group housing cages described above. Birds were visually assessed for health at least two times per day by the researcher (LMG) and one additional time per day by the animal care staff. All birds were between one and three years of age at time of testing. The work described here was conducted with the approval of the University of St Andrews Animal Welfare and Ethics Committee.

### 2.2 Apparatus

**Fig. 1:**
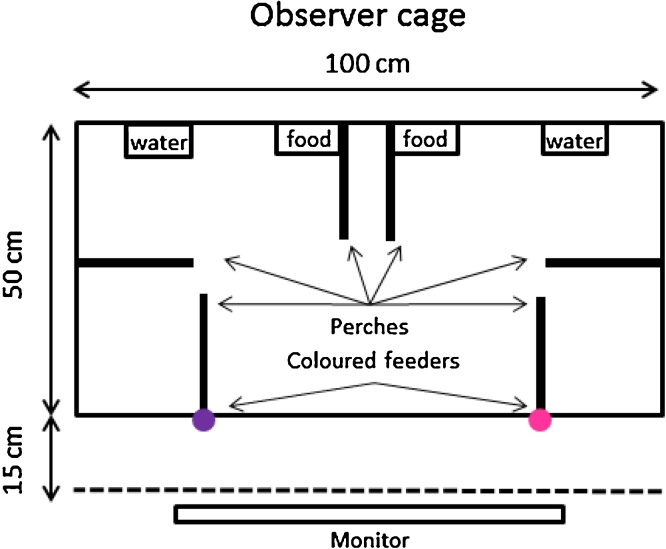
Scale drawing of the top down view of the observer cage. The dashed line represents a white opaque barrier between the observer cage and the video screen that was in place at all times except during the observation phase. The food bowls were removed from the observer cage 2 h prior to the start of the observation phase. The coloured feeders were present for the observer only during the test phase. The demonstrator cage (not pictured) was identical to the observer cage, with one exception: the food bowls were never present and the baited colour feeder was the only food source available to the demonstrator.

The experiments were carried out in a test room that contained a demonstrator cage and four observer cages. White opaque curtains between each cage allowed subjects to hear and vocally interact but not see each other for the duration of the experiments. Each observer cage (100 × 50 × 50 cm see Fig. 1) contained two water bowls, a cuttlefish bone and a vitamin block and six perches and two grey food dished on the opposite side of the cage from where the experimental feeders were located. During the observation and subsequent test phase (described below) coloured feeders (one pink, one purple, wrapped in coloured opaque paper) were attached to each cage. A video screen (ViewsSonic Thin Film Transistor, model # VS15804) situated 15 cm from the long side of the observer cage was concealed by a white opaque curtain. The demonstrator cage was identical to that of the observers’, with the exception of the grey food bowls as the baited colour feeder was the only food source for the demonstrator for the duration of the experiment. Each cage contained three bird box cameras (SpyCameraCCTV, Bristol, UK) connected to a laptop computer. A GoPro Hero3 (GoPro, Incorporated, California, USA) recorded the behaviour of the demonstrator and was used to live stream the demonstrator in Experiment 1. The mini-HDMI connection from the GoPro was connected to a 1 × 4 HDMI Splitter (HDelity by Cablesson^®^), which connected to each of the four video screen via a separate HDMI cable.

### 2.3 Experiment 1: live streaming video demonstration

#### 2.3.1 Subjects

The subjects were 43 adult zebra finches (16 females, 26 males) that were bred at the University of St Andrews. Four observers were tested in each trial (4 observers per demonstrator) and there were two experimental groups: *group one,* female observers, and *group two,* male observers. The demonstrators were always male. For *group one* there were two demonstrators, each used in two trials, acting as demonstrator for the pink feeder in one trial and the purple feeder in the second trial (*n* = 16 observers). For *group two* there were five demonstrators. Four demonstrators participated in one trial each and one demonstrator participated in two trials (one pink, one purple, *n* = 24 observers).

#### 2.3.2 Procedure

Each trial lasted approximately 24 h. The observer viewed the behaviour of the demonstrator in the same room, visually occluded from the observer with white opaque curtains, via a video screen. The images that the observer viewed on the video screen were streamed live via the GoPro (30fps 1080p). While the demonstrator could not see the observers he could vocally interact with them.

On Day 1, one bird was placed in each of the observer and the one demonstrator cage. At this time, all opaque curtains between all birds, and between the observer and the video screen were in place so the birds could not see one another or the video screen. The only food available to the demonstrator bird was provided in one of two experimental feeders (pink or purple). Thus the demonstrator bird learned which feeder to ‘demonstrate’ during the observation phase (described below) the next day. On Day 2, food was removed from all cages (4 observers and 1 demonstrator) two hours post light onset.

There were two phases in each trial, the observation phase followed by a test phase. The observation phase began after the two-hour food deprivation period. During the observation phase, the feeder that had been removed from the demonstrator’s cage during the food deprivation period was returned. Now the demonstrator had two feeders, one pink, one purple, only one of which contained seeds. The 30-min observation phase started when the opaque curtain between the observer cages and the video screen was removed so that the observer could view the demonstrator’s behaviour on the video screen.

During the test phase, which began immediately after the 30-min observation phase, the opaque barrier between the observer and the video screen was returned and one pink and one purple feeder, each containing seeds, was attached to the observer cage. With the exception of the demonstration that was just viewed, these coloured feeders were novel to the observers. The spatial location of the pink and purple feeders on the observers’ cage mirrored that of the video image of the demonstrator. In this way, both colour and spatial cues indicate the demonstrator’s feeder choice. This test phase lasted 60 min. The behaviour of the observers was video recorded. At the end of the test phase, the observers were returned to their stock cages, and new birds were placed in the observer cages for testing on the following day.

### 2.4 Experiment 2: video playback demonstration with sound

#### 2.4.1 Subjects

The subjects in this Experiment were 16 adult female zebra finches, seven were bred at the University of St Andrews and nine were from a local breeder.

#### 2.4.2 Demonstrator videos

Thirty-minute video recordings of the demonstrators for *group one* from Experiment 1 were used as playbacks for the remainder of Experiments (2–4). In Experiment 2 these videos were played on the same video screens with full audio (measured at 60 dB at head height of the zebra finch when perched on the perches closest to the video monitor.). There were four videos total: each male served as a demonstrator on two trials (4 observers per trial), demonstrating a different colour each trial.

#### 2.4.3 Procedure

The procedure for this experiment was similar to the Experiment 1 with the following exceptions. Here, the video on the screen was a playback and was not live streamed. Thus, these observers saw and heard the demonstrators (same as those in Experiment 1) but they could not vocally interact with him because the video was pre-recorded.

### 2.5 Experiment 3: video playback demonstration with no sound

#### 2.5.1 Subjects

The subjects were 16 adult female zebra finches, nine were bred at the University of St Andrews and seven were from a local breeder.

#### 2.5.2 Procedure

The procedure for this experiment was similar to that of Experiment 2 except that the demonstrator videos were played without sound.

### 2.6 Experiment 4: video playback demonstration with no sound and live male decoy

#### 2.6.1 Subjects

The subjects were 23 adult zebra finches (20 females, 3 males) bred at the University of St Andrews. Two decoy males were used for two trials each and a third decoy male was used for one trial (*n* = 20 observers total).

#### 2.6.2 Procedure

The procedure for Experiment 4 was similar to Experiment 3 except that there was a live male in the room who was not visible to the observers. Thus, the observers could vocally interact with a male but he was not the demonstrator they could see in the video. See Table 1 for breakdown of which types of visual and vocal information were available to the observers across the four Experiment groups.

### 2.7 Scoring

From the video recordings of each trial the number of pecks delivered to each feeder were counted using Solomon Coder (beta version 16.06.26). To quantify feeder colour preference the following measures were calculated: (1) proportion of pecks to the feeder containing seeds for the demonstrator bird and, (2) the proportion of pecks by the observer bird to the demonstrator’s feeder colour.

### 2.8 Demonstrator performance

There were two male demonstrators for *group one*, each participated in two trials (*n* = 4 observers per trial). Demonstrator A pecked the baited feeder 944 times when that feeder was purple and 1388 times when that feeder was pink. Demonstrator B pecked the baited feeder 892 times when that feeder was purple and 753 times when that feeder was pink. No pecks were ever delivered to the non-baited feeder. Videos of these four trials were used during the observation phase for Experiments 2–4. Thus, the female observers across all of these Experiments saw the same visual demonstration.

There were five male demonstrators for *group two.* Demonstrator A was used in two trials: he did not feed during the observation on one trial and thus the four observers for this trial were not used. In his second trial, Demonstrator A pecked at the baited feeder (pink) 522 times. Demonstrator B did not feed during the observation phase and thus the four observers for this trial were not used. Demonstrator C pecked at the baited feeder (purple) 180 times. Demonstrator D pecked at the baited feeder (pink) 121 times. Demonstrator E pecked at the baited feeder (purple) 114 times.

### 2.9 Statistical analysis

Binomial test were conducted to determine if the proportion of pecks for each observer differed significantly from no-preference (i.e. 0.5). Each observer birds could therefore be classified as having (1) copied the colour choice of the demonstrator bird, (2) avoided the colour choice of the demonstrated bird, or (3) having no preference. A Pearson Chi-square test was used to compare the proportion of females who copied across the four experiments. To test for systematic copying in the Experiments, we carried out one-sample Wilcoxon signed-rank tests on the proportion of pecks by the observer bird to the colour of feeder used by the demonstrator. For all but *group two* in the Experiment 1 (the male observers) we used one-tailed tests because we had *a priori* expectations that if video demonstration worked in our experimental contexts, there should have been an effect in the same direction as for live demonstrators (Guillette and Healy, 2014). For *group two* in the Experiment 1 we used a two-tailed test because male observers were not expected to copy the foraging decision of male demonstrators (Guillette and Healy, 2014). All the data we report are mean ± standard error.

## 3 Results

### 3.1 Individual performance

Ten observers did not eat during the test phase leaving the final group totals: Experiment 1 males, n = 15, females, n = 15; Experiment 2, n = 15; Experiment 3, n = 15; Experiment 4, n = 14. The number of pecks by all observers ranged from 9 to 1005. All but two birds (females in Experiment 2, *Z* = 0.1*, P* = 0.4 and Experiment 4, *Z* = 0.78*, P* = 0.29) preferred one colour feeder over the other. Forty-nine (of 72) birds’ preference scores differed significantly from 0.5 (all *Z*’s >│2.92│, *P*’s < 0.006). The binomial test could not be performed on the remaining 23 birds because these individuals exclusively ate from one feeder colour (i.e. preference = 1.0) so the performance of these individuals was considered to be different from that of chance. Overall, birds chose the feeder of the same colour as that used by the demonstrator (Wilcoxon signed-rank test, *W* = 1794, *N* = 74, *P* = 0.014).

### 3.2 Group performance

**Fig. 2:**
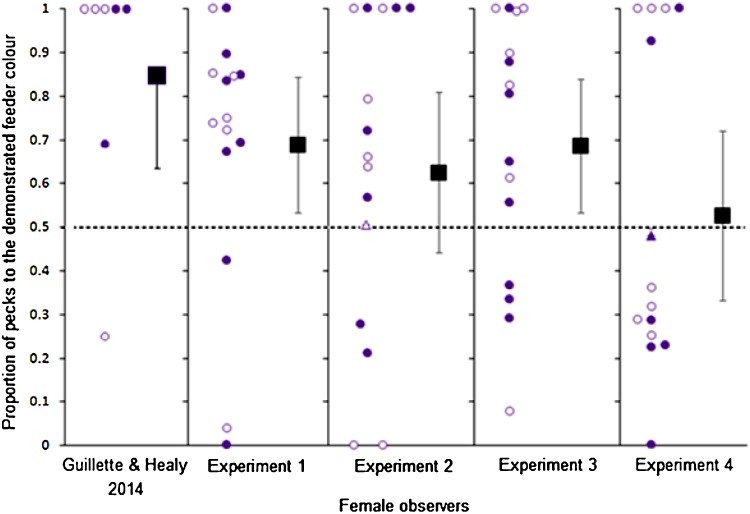
The proportion of pecks (y-axis) to the demonstrated colour feeder for female observers across the four experimental groups and live demonstrations from Guillette and Healy 2014 (*x*-axis). Filled circles represent choices of the observer when their demonstrator fed from the purple feeder and open circles represent when the demonstrator fed from the pink feeder. The square represents the mean proportion of each group ± 95% confidence intervals and the triangles represent individual with no preference for either feeder. Guillette and Healy (2014) used a live demonstrator, Experiment 1 used live streaming demonstration, Experiment 2 used video demonstration with audio, Experiment 3 used video demonstration without audio, Experiment 4 used video demonstration without audio and a live decoy male present (auditory only). The dashed horizontal line represents no preference between the demonstrated and non-demonstrated options. The demonstrators were male and the same videos were used in all experiments. The data from Guillette and Healy (2014) are reprinted from *Behavioural Processes, 108,* 117–182, Mechanisms of copying in zebra finches, with permission from Elsevier.

**Fig. 3:**
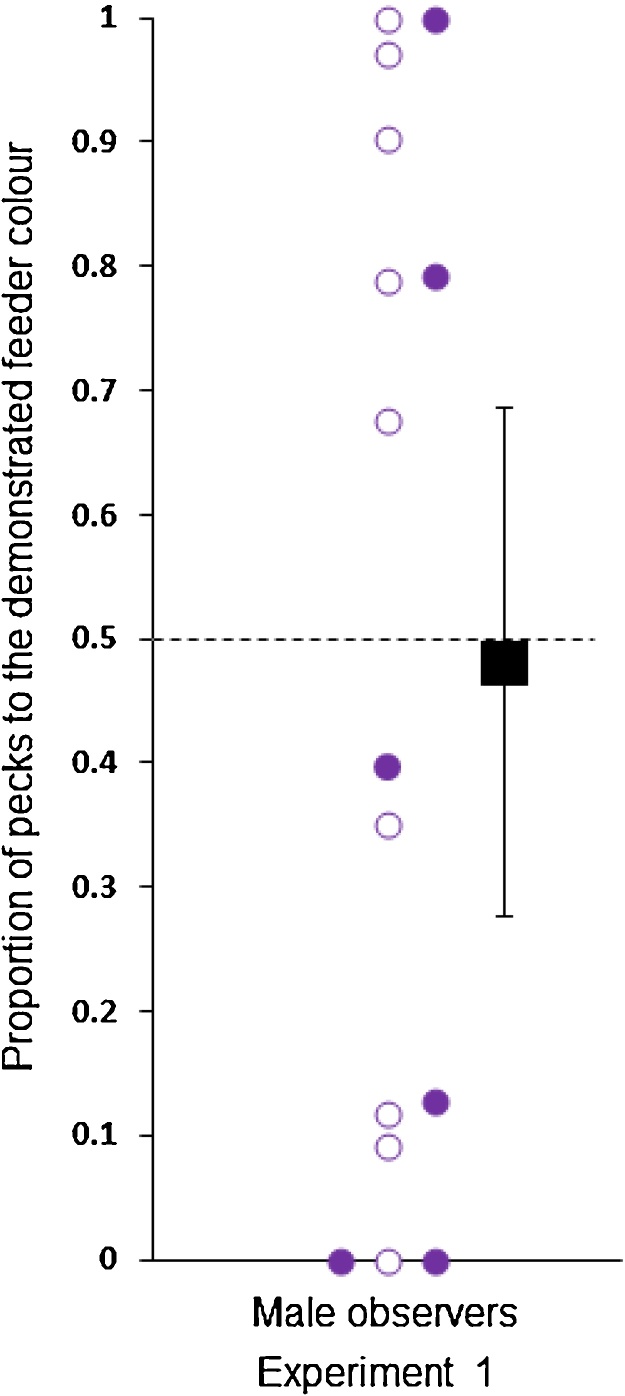
The proportion of pecks (y-axis) to the demonstrated colour feeder made by the male observers in Experiment 1 (*x*-axis). Filled circles represent those observers that watched a demonstrator feed from the purple feeder and open circles represent the observers that watched a demonstrator feed from the pink feeder. The dashed horizontal line represents no preference between the demonstrated and non-demonstrated options. The square represents the mean proportion of each group ± 95% confidence intervals.

Using these dichotomous data (copy versus avoid), we found that preferences for the demonstrated colour did not differ across the five groups (Pearson *X^2^* (4), *N* = 72) = 8.0, *P* = 0.09. The females that observed the live streaming video demonstrator (Experiment 1) were much more likely to direct their pecks to the feeder of the demonstrated colour than expected by chance (0.69 ± 0.08, Wilcoxon signed-rank test, *Z* = 1.88, *N* *=* 15, *P* *=* 0.03; Fig. 2). Eleven of 15 female observers pecked more at the feeder of the demonstrated colour than to the feeder of the other colour. The males that observed the live streaming video demonstrator (Experiment 1) were no more likely to direct their pecks to the feeder of the demonstrated colour than expected by chance (0.48 ± 0.10, Wilcoxon signed-rank test, *Z* = −0.23, *N* *=* 15, *P* *=* 0.82; Fig. 3). Seven of 15 male observers pecked more at the demonstrated feeder than at the other feeder.

The females that observed video playback with sound (Experiment 2) did not direct more of their pecks to the feeder of the demonstrated colour than expected by chance (0.62 ± 0.09, Wilcoxon signed-rang test, *Z* = 1.24, *N* *=* 15, *P* *=* 0.11; Fig. 2). Ten of 15 of the female observers pecked more at the demonstrated colour than to the non-demonstrated colour feeder and one observer had no preference.

In Experiment 3 (video demonstrations without sound), the females were more likely to direct their pecks to the feeder of the demonstrated colour than to the feeder of the other colour (0.69 ± 0.08, Wilcoxon signed-rang test, *Z* = 1.99, *N* *=* 15, *P* *=* 0.02; Fig. 2). Eleven of 15 female observers pecked more at the demonstrated colour feeder.

In Experiment 4 (video playback without sound with a live decoy male) did not direct more of their pecks to the feeder of the demonstrated colour than to the feeder of the other colour (0.53 ± 0.10, Wilcoxon signed-rank test, *Z* = 0.28, *N* *=* 14, *P* *=* 0.39; Fig. 2). Five of 14 female observers pecked more at the demonstrated colour feeder and one observer had no preference.

## 4 Discussion

Zebra finches used social information about novel foraging decisions when the information was live-streamed to them on a video screen as when the information is delivered by live demonstrators (Experiment 1; Guillette and Healy, 2014): female zebra finches copied the foraging decision of males but males did not. These results are also consistent with similar work on social learning in zebra finches using a two-demonstrator paradigm to determine *who* the birds copy. In these studies, birds chose between the social information from two distinct demonstrators and while females copied the foraging decisions of males (over females) males did not systematically copy either sex (Benskin et al., 2002, Katz and Lachlan, 2003). Familiarity between male observers and demonstrators may be key for social information use as although males in the current experiment also did not copy the foraging decisions of an unfamiliar male demonstrator, but males did copy the foraging decisions of a familiar male over an unfamiliar male in a two-demonstrator test (Benskin et al., 2002). In addition, males copy nest material choices of familiar but not unfamiliar males (Guillette et al., 2016). Familiarity is not the whole story, however, as males copy the foraging decisions unfamiliar males reared in large-broods over those made by unfamiliar males reared in small broods (Riebel et al., 2012).

Our data show that in addition to factors, such as brood size or colour of leg band of the demonstrator (Benskin et al., 2002, Riebel et al., 2012), the apparent attention of the demonstrator towards the observer is also relevant to copying by female observers. Although performance across the four experimental groups was not different, there was systematic copying in some but not all of the contexts. For instance, female zebra finches did not copy male demonstrators when they could see and hear him but not vocally interact with him. They also did not copy when they could see a male demonstrator but could vocally interact another male during the demonstration. In the absence of sound from the male demonstrator, however, the females did copy. Therefore, while vocalizations by the demonstrator are not necessary for copying, they can reduce the probability of social learning.

The impact of the presence/absence of vocalizations in video playback experiments of social learning appears to be species specific. Unlike zebra finches, for example, budgerigars and Burmese red jungle fowl copy video demonstrators that ‘ignore’ them (i.e. video playback with sound; McQuoid and Galef, 1993, Mottley and Heyes, 2003). Indeed, the non-interactive vocalizations of the demonstrator jungle fowl during the ‘ignore’ condition were necessary for the transfer of social information: jungle fowl observers did not copy the demonstrator if the video was silent (McQuoid and Galef, 1993), whereas zebra finches in the current experiment did copy under these conditions (this ‘silent’ condition was not tested with budgerigars). The explanation for this interspecific variation is not clear although ecology does not seem to hold the answer as both budgerigars and zebra finches, at least, are both highly social and live in flocks that range from around 20 to over a few hundred individuals. They also both originate from the dry areas in Australia and form strong pair bonds remaining in close contact with their mates throughout the year (Brockway, 1964, Zann, 1996).

Whether or how they learn their vocalizations also does not seem to explain the variation. The zebra finch is a close-ended learner, while the budgerigar is an open-ended learner and the jungle fowl is not a vocal learner at all, although it does have a referential alarm call to communicates information about the size, speed, and distance of avian predators (Wilson and Evans, 2012). Determining why zebra finches are more sensitive to, or pay more attention to vocal information, than do either budgerigars or red jungle fowl, even when the information they are copying is visual needs further investigation.

In the current experiment zebra finches did not copy the demonstrator when there was a mis-match between vocal and visual information (video without audio, live, out of sight male). It seems plausible that the females failed to copy because as they swapped vocalizations with the live male, they did not pay attention to the video. This supposition is supported by female zebra finches preferring their own mate over an unknown male when vocal and visual cues match but she does not prefer her own mate when his vocalizations are replaced by those of unknown individual (Galoch and Bischof, 2006). Although the birds in our experiments were not mates, the mis-match in vocal communication appears to have been sufficient to disrupt the female’s acquisition or use of social information from the male she would otherwise have copied. Taken together, the current results point to a special and important role not for vocal information *per se* but rather for appropriate reciprocity when vocal information is provided.

It is unclear whether the females that were ignored by the male demonstrator (1) did not acquire the social information or (2) did not use the social information. This distinction was discernible in inexperienced male zebra finches watching experienced males build a nest. In that experiment, only those males that saw a familiar male building copied the demonstrator’s material choice, whereas the males that saw an unfamiliar male building did not copy the choices of their demonstrator (Guillette et al., 2016). And yet, the inexperienced males that saw an unfamiliar male building did touch and interact with the demonstrated material first, which shows that they had acquired the social information, even though they did not use that information when they came to build their own nest.

Even in this social species, then, the circumstances under which the animals use the information they acquire from conspecifics is much more varied than one might have expected and may be due, in part, to individual differences in sampling behaviour (Rosa et al., 2012). This would suggest that although we have a broad canvas for the contexts for social learning (Laland, 2004) we are not yet in a position to either predict or to explain the empirical outcomes of social learning experiments.

## Figures and Tables

**Table 1:** Breakdown of the type of visual and vocal information that were available to the observers in the four Treatment groups in the current experiment and from one condition in Guillette and Healy 2014. Matched refers to congruency between the information types and mis-matched indicates in congruency between the visual and vocal information.

	Guillette and Healy (2014)	Treatment 1	Treatment 2	Treatment 3	Treatment 4
	Live demonstrator: face-to-face with observer	Streaming video in real time: demonstrator present but visually isolated from observers	Video playback with sound: ignore condition	Video playback without sound: silent condition	Video playback without sound + decoy male
Visual information	interactive	non-interactive: video screen	non-interactive: video screen	non-interactive: video screen	non-interactive: video screen
Vocal information	interactive	interactive	non-interactive: audio track from video playback	No audio information	interactive with decoy male present but visually isolated from observers.
Interaction between visual & vocal informtion	matched	matched	matched	NA − no vocal information	mis-match
